# Dynamic Characteristics and Damage Constitutive Model of Mudstone under Impact Loading

**DOI:** 10.3390/ma15031128

**Published:** 2022-01-31

**Authors:** Ruihe Zhou, Hua Cheng, Haibing Cai, Xiaojian Wang, Longhui Guo, Xianwen Huang

**Affiliations:** 1 School of Civil Engineering and Architecture, Anhui University of Science and Technology, Huainan 232001, China; zrhaust@163.com (R.Z.); haibingcai@163.com (H.C.); xjwangyhl@163.com (X.W.); guolonghui7864@163.com (L.G.); huangxianwen194@163.com (X.H.); 2 School of Resources and Environmental Engineering, Anhui University, Hefei 230022, China

**Keywords:** mudstone, impact test, strain rate, dynamic strength criterion, dynamic damage constitutive model

## Abstract

The mechanical response characteristics of mudstone from the ingate roadway of the west ventilation shaft in Yuandian No. 2 coal mine, Huaibei City, Anhui Province, China to dynamic loads were quantified in single- and cyclic-impact compression tests, using the split-Hopkinson pressure bar test device. The dynamic stress–strain relationships and the failure characteristics of mudstone samples under different impact loads were analyzed systematically. Considering the “rate effect” of the mudstone dynamic strength, the dynamic strength criterion of mudstone was proposed, and the dynamic damage constitutive model of mudstone was established, based on the statistical damage theory. In response to single-impact loads, with increasing impact pressure, the mudstone peak stress and strain gradually increased, and the peak stress and average strain rate increased nonlinearly. In response to cyclic-impact loads, with an increasing number of impacts, the mudstone peak stress first increased and then decreased, and the peak strain increased gradually. With increasing impact pressure, the number of impacts to the samples’ failure decreased gradually. By parameter identification and comparative analysis of the test results, the proposed dynamic damage constitutive model of mudstone was validated. The model can be used for stability analysis of roadway-surrounding rock under dynamic loads.

## 1. Introduction

Recently, with deeper coal mining, the instability of soft rock roadways with difficult support has been increasing [1,2]. Studies suggest that the stability of soft rock roadways depends on the in situ stress, lithology, support form, mining, and other factors, while the blasting dynamic load owing to drilling and blasting construction of soft rock roadways also significantly affects the surrounding rock stability [3,4,5,6]. For example, the ingate and connecting chamber groups of deep coal mines are mostly located in the coal measure stratum with interbedded sandstone, argillaceous sandstone, and mudstone. During the chamber group construction, the dynamic load owing to drilling and blasting increases the frequency and intensity of repeated disturbances to its surrounding rock, endangers the stability of the surrounding rock, and occasionally destabilizes the surrounding rock, leading to support failures [7,8,9]. Therefore, studying the dynamic characteristics of mudstone in response to impact loads is important for formulating corresponding damping technical measures, improving the impact of drilling and blasting construction dynamic loads on the surrounding rock stability of soft rock roadways in deep coal mines, and ensuring the roadway support safety.

The strength of rock under dynamic loads is significantly correlated with the strain rate [10,11,12]. Scholars worldwide have performed theoretical and experimental studies on the dynamic characteristics of hard rock types such as sandstone and granite under blast loading and impact loading, obtaining significant insights. For the research on the dynamic characteristics of rock under blast loading, Xu et al. [13] studied the dynamic response of deep rock mass under blast loading, considered that deep rock mass is more vulnerable to damage than shallow rock mass under blast loading, and put forward a new safety criterion for blasting vibration of deep rock mass. Yilmaz et al. [14] studied the behavior of rock mass under blast loading by using three-dimensional finite difference numerical modelling, and proposed the dynamic compressive strength equation of rock material under blast loading.

For the research on the dynamic characteristics of rock under impact loading, Mishra et al. [15] analyzed the mechanical response law of rock tunnel under impact loading by using the finite element software. Chakraborty et al. [16] studied the dynamic stress–strain response, peak stress, elastic modulus, and force equilibrium at the incident and transmission bar ends of three Himalayan rocks under impact loading by using a split-Hopkinson pressure bar. Li et al. [17] conducted uniaxial compression tests on green sandstone under repeated impact loads and studied the deformation characteristics and microstructural evolution characteristics of green sandstone. Ping et al. [18] carried out impact compression tests on coal-mine sandstone samples under different loading rates, and the analysis showed that the dynamic compressive strength, dynamic elastic modulus, and strain rate of sandstone were positively correlated with the incident energy. Braunagel et al. [19] used an improved split-Hopkinson compression bar to study the effect of cyclic-impact loads on the dynamic compressive strength of westerly granite. The dynamic strength and deformation characteristics of rock under impact loading can be comprehensively described using a dynamic constitutive model. Yu et al. [20] established a dynamic constitutive equation of rock based on the modified overstress model, which can better describe the dynamic mechanical behavior of rock under impact loading. Shan et al. [21] considered the influence of the rock dynamic deformation process on its mechanical parameters, and a time-dependent damage constitutive model for the dynamic failure of rock was established by combining a statistical damage model with a viscoelastic model. Xie et al. [22] considered the influence of the material damage under dynamic loads, and established a simplified constitutive equation of the damage overstress model based on a dimensional analysis method. Yang et al. [23,24,25,26] established a rock dynamic damage model by the parallel connection of an elastic damaged body and viscous body based on the rock static Drucker–Prager criterion and viscoplastic theory. In summary, up to the present, research on the mechanical properties of rock mass under cyclic-impact loads has mainly focused on granite, sandstone, and other hard rock types, while relatively little attention has been given to the coal-mine mudstone.

Therefore, taking the mudstone at the floor of the ingate horizontal roadway in the west ventilation shaft of the Yuandian No. 2 mine as the research object, a single-impact test under different impact pressures and a cyclic-impact test assuming a fixed impact pressure were carried out on the mudstone samples, using the split-Hopkinson pressure bar (SHPB) test device. Considering the rate correlation of the dynamic deformation process, the rock dynamic strength criterion was established, and the dynamic damage constitutive model of mudstone under different impact loads was established based on the statistical damage theory, to improve the theory and the method of rock dynamic deformation process simulations.

## 2. Impact Compression Test of Mudstone

### 2.1. Preparation and Microanalysis of Mudstone Samples

#### 2.1.1. Sample Preparation

**Figure 1:**
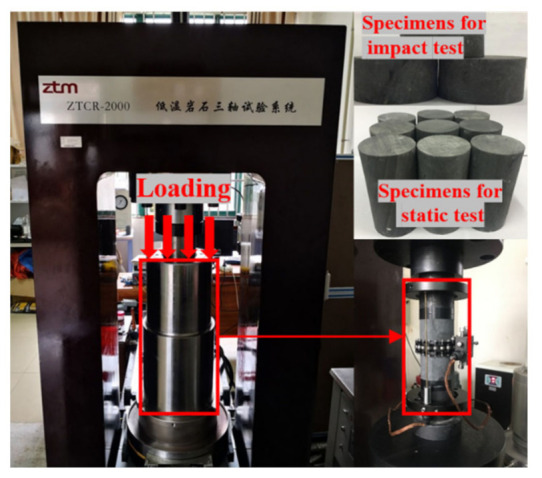
Mudstone samples and the static load test device.

The rock samples were extracted from the mudstone of the Permian Upper Shihezi formation at the 431.8 m depth of ingate in the west ventilation shaft of Yuandian No. 2 mine, Huaibei City, Anhui Province. The sizes and the test processes of the extracted rock samples were in accordance with the standards of engineering rock mass test methods (GB/T 50266-2013). As shown in Figure 1, the rock triaxial test sample was cylindrical (diameter, 50 mm; height, 100 mm), and the split-Hopkinson Pressure Bar (SHPB) impact test sample was cylindrical as well (diameter, 50 mm; height, 25 mm). The measured basic mechanical parameters of mudstone were as follows: the average unit weight was 2590 kg/m^3^, the average longitudinal wave velocity was 3820 m/s, and the uniaxial compressive strength was 35.85 MPa.

#### 2.1.2. Microstructure and Mineral Composition of Mudstone Samples

**Figure 2:**
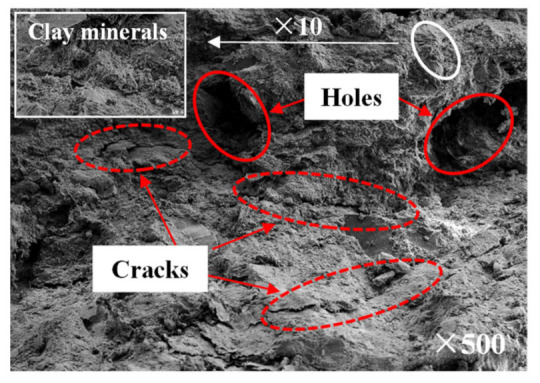
SEM image of a mudstone sample.

**Figure 3:**
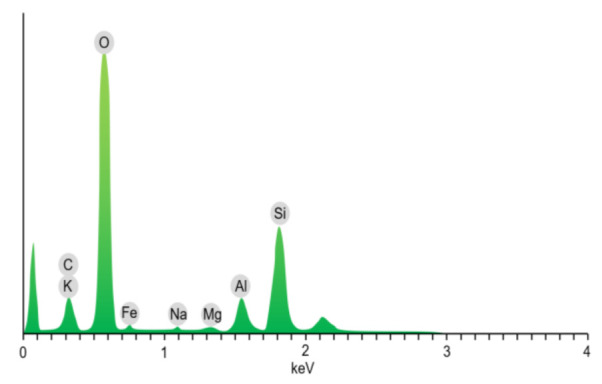
EDS results for mudstone samples.

**Figure 4:**
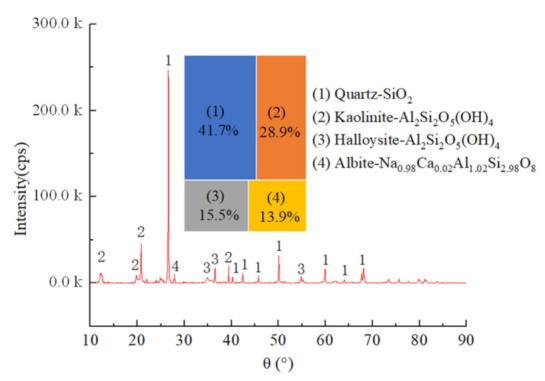
XRD diffraction pattern of mudstone.

The microstructure and mineral composition of the mudstone samples were examined using scanning electron microscopy (SEM, Hitachi high-resolution cold field emission scanning electron microscope Regulus8100, Tokyo, Japan), energy-dispersive X-ray spectroscopy (EDS, Hitachi high-resolution cold field emission scanning electron microscope Regulus8100, Tokyo, Japan), and X-ray powder diffraction (XRD, Rigaku Corporation smartlab-9kw rotating target X-ray powder diffractometer, Tokyo, Japan), respectively. The test and analysis results showed that the particle sizes of the mudstone samples were different, and flocculent cements dominated by clay minerals were attached to the particles’ surface. Mudstone particles in general exhibited accumulation, with many microscopic defects such as cracks and holes (Figure 2). The main elements in the samples were Si, Al, Na, and O (Figure 3). The main components of the mudstone samples were quartz, kaolinite, halloysite, and albite, among which quartz accounted for approximately 41.7%, kaolinite accounted for approximately 28.9% of the total composition, and the proportions of halloysite and albite were 15.5% and 13.9%, respectively (Figure 4).

### 2.2. Test Device and Scheme

**Figure 5:**
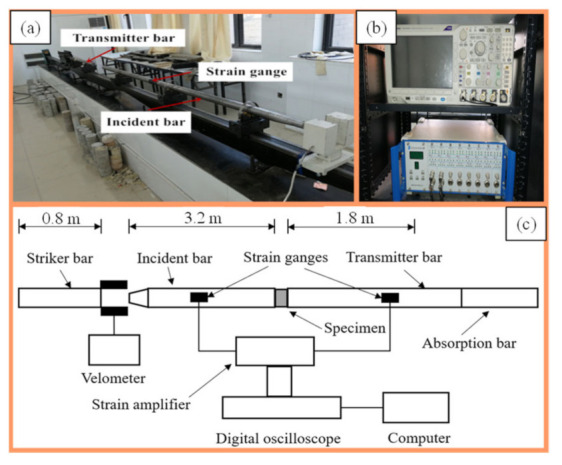
SHPB test device system. (**a**) Test equipment; (**b**) Monitoring equipment; (**c**) Test principle.

The SHPB test device of the Impact Dynamics Laboratory of the Anhui University of Science and Technology in Huainan City, Anhui Province, China was used for testing the dynamic characteristics of the mudstone samples (Figure 5). The diameter of the SHPB device’s pressure bar was 50 mm, the punch was a spindle, the impact waveform was rectangular, the pressure bar and the punch were made of alloy steel (density, 7800 kg/m^3^; elastic modulus, 210 GPa; longitudinal wave velocity, 5190 m/s). The data acquisition system consisted of a strain gauge (Aifute SDY2107A ultra-dynamic strain gauge, Qinhuangdao, China) and an oscilloscope (Yokowaga-DL850E oscilloscope, Tokyo, Japan).

The mudstone samples were divided into two groups, for single-impact tests and cyclic-impact tests at the same impact pressure (impact repeated in triplicate, same loading conditions). Five different impact pressures of 0.2, 0.25, 0.3, 0.4, and 0.6 MPa were used in the single-impact load tests. According to the pre-test results, it is found that 0.2 MPa is a threshold value of test block impact. When this load is exceeded, the sample will appear obvious cracking or crushing under single impact, and the second impact cannot be repeated. Hence, three different fixed impact pressures of 0.15, 0.175, and 0.20 MPa were used in the cyclic-impact pressure tests for better experimental results.

## 3. Test Results and Discussion

### 3.1. Test Results

Combined with the waveform characteristics obtained from the mudstone SHPB impact tests, the “simplified three wave method” [27] was used for processing the test data. The “simplified three wave method” is a calculation method combining the respective advantages of the “three wave method” and the “two wave method”, that is, the “three wave method” is used to calculate the strain rate and strain, and the “two wave method” is used to calculate the stress. Table 1 and Table 2 list the test results for the samples subjected to single- and cyclic-impact loads, respectively.

### 3.2. Stress–Strain Curve Analysis

#### 3.2.1. Single-Impact Test, Different Impact Pressures

**Figure 6:**
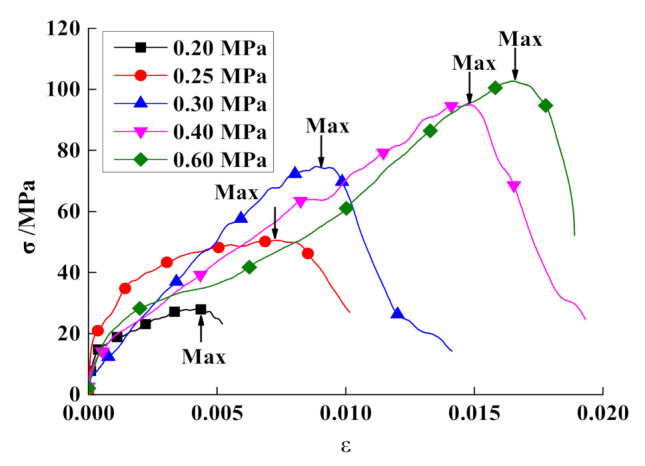
Stress–strain curves of mudstone samples, for different impact pressure.

The stress–strain curves for the mudstone samples subjected to single impacts at different impact pressures are shown in Figure 6. Evidently, with increasing impact pressure, the peak stress and peak strain of the mudstone samples gradually increased, the peak stresses are 28.17 MPa, 50.53 MPa, 74.75 MPa, 94.95 MPa, and 102.69 MPa, respectively. At the impact pressure of 0.6 MPa, the dynamic peak stress of the mudstone samples was approximately 2.86 fold the uniaxial compressive strength of the mudstone samples subjected to static loads. This phenomenon occurred because, compared with the static-load results, many microscopic cracks were formed in the samples subjected to impact loads, and the deformation and the energy absorbed and dissipated by the samples increased significantly, increasing the peak stress of the samples relative to the static-load results.

The dynamic stress–strain curve of a mudstone sample can be roughly divided into three regions: (1) the linear elastic stage; (2) the plastic deformation stage; and (3) the failure stage. In the elastic stage, the stress increases linearly with increasing strain. Under an impact load, the microscopic cracks in the sample continue to sprout and develop. The plastic deformation stage occurs after reaching the elastic limit, and the slope of the curve gradually decreases to zero. When the peak stress is reached, the internal cracks in the stressed sample expand, forming a macroscopic fracture surface. The strain increase in the sample is small, the stress decreases sharply, and the mudstone sample enters the failure stage.

**Figure 7:**
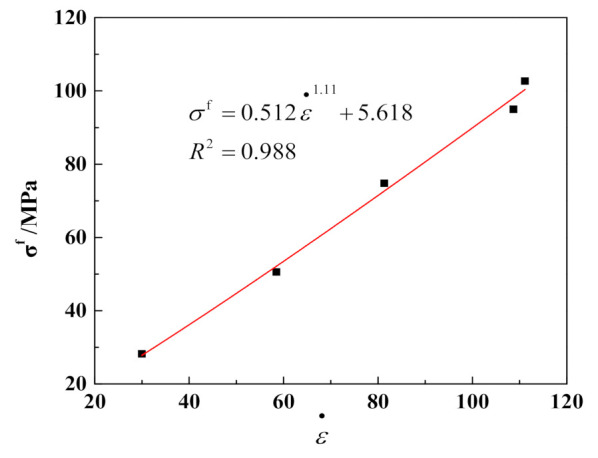
Relationship between peak stress and average strain rate.

The relationship between the peak stress and the average strain rate for the mudstone samples subjected to single impacts, for different impact pressures, is shown in Figure 7. The average strain rate is the average of all strain rates on the strain rate versus time curve. Evidently, the peak stress of mudstone increases gradually with increasing the average strain rate, which is manifested as an exponential relationship, indicating that the dynamic strength of rock has an obvious rate correlation and is nonlinearly positively correlated to the strain rate.

#### 3.2.2. Cyclic-Impact Test, Fixed Impact Pressure

**Figure 8:**
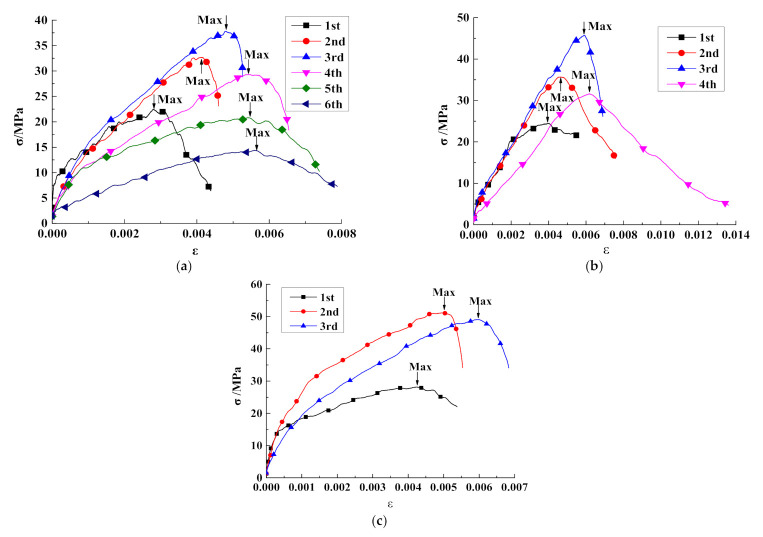
Stress–strain curves for the mudstone samples subjected to the cyclic-impact loading: (**a**) 0.15 MPa; (**b**) 0.175 MPa; (**c**) 0.20 MPa.

The stress–strain curves for the mudstone samples subjected to cyclic impacts are shown in Figure 8. Comparing the cyclic-impact times for the mudstone samples subjected to different impact pressures, it is observed that the number of impacts until failing the sample decreases gradually with increasing impact pressure, which are 6 times, 4 times, and 3 times, respectively. Compared with the dynamic stress–strain curves for the mudstone samples subjected to the same impact pressure, as the number of impacts increases, the peak stress of mudstone increases first and then decreases, while the peak strain increases gradually.

As shown in Figure 8a, at the impact pressure of 0.15 MPa, the dynamic mechanical characteristics of mudstone exhibit “a strengthening effect combined with a softening effect” [28]. The peak strength reaches a maximum after the third impact, and then the “softening effect” of mudstone becomes more obvious with increasing the number of impacts. As shown in Figure 8b, at the impact pressure of 0.175 MPa, the dynamic mechanical characteristics of mudstone exhibit an obvious “strengthening effect”. During the first three impacts, the peak strength increases gradually until it reaches a maximum, and the strength reduction in mudstone during the fourth impact shows a “softening effect”. As shown in Figure 8c, at the impact pressure of 0.20 MPa, the “strengthening effect” of mudstone becomes more obvious in the process of three impacts.

**Figure 9:**
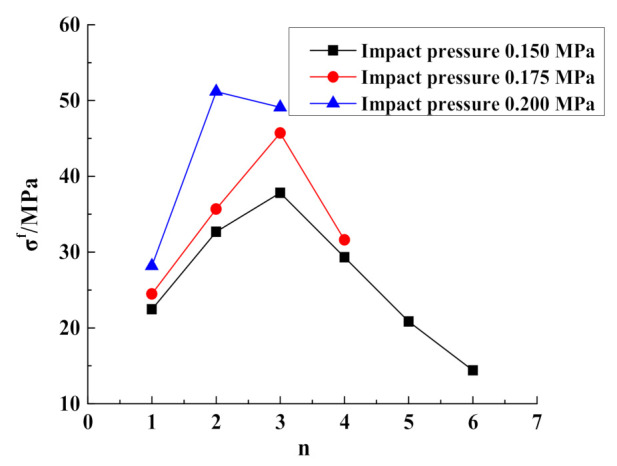
Peak stress vs. the number of cyclic impacts, for different impact pressures.

Figure 9 shows the relationship between the cyclic-impact time and peak stress, for the studied mudstone samples, for three different impact pressures. Evidently, the peak stress first increases and then decreases with increasing the number of impacts. At the impact pressure of 0.15 MPa, owing to the low impact pressure, no serious damage is inflicted on the sample after the first impact, the compaction is dominant, and the microscopic cracks inside the sample are compacted, which increases the mudstone strength. The peak stress of the mudstone following the second impact is much higher than that following the first impact. The peak stress of mudstone reaches a maximum after the third impact. As the number of impacts continues to increase, the peak stress decreases slowly and reaches a minimum after the sixth impact. Analysis suggested that, after each impact load, part of the energy is applied toward aggravating the damage to the sample; thus, the peak strength gradually decreases. As the number of impacts increases, more microscopic cracks are generated in the sample, and the continuous accumulation of internal damage leads to a continuous decrease in the rock bearing capacity. After a certain number of impacts, the peak stress of the mudstone sample suddenly decreases, which is manifested as the deterioration of its bearing capacity.

### 3.3. Failure Morphology of Samples under Impact Loads

#### 3.3.1. Single-Impact Scenario, Different Impact Pressures

**Figure 10:**
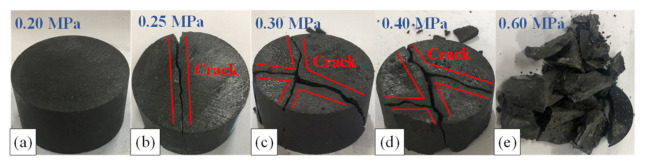
Failure morphology of the mudstone samples subjected to single-impact loads. (**a**) 0.20 MPa; (**b**) 0.25 MPa; (**c**) 0.30 MPa; (**d**) 0.40 MPa; (**e**) 0.60 MPa.

The failure morphology of the studied mudstone samples subjected to a single impact is shown in Figure 10, for different impact pressures. For low impact pressures, the sample deformation is small, and the samples are in the state of large block crushing. As the impact pressure increases, the crushing range of the sample gradually expands from the edge to the center, the crushing degree of the sample increases, from large block crushing to small block crushing, the number of fragments increases, and the volume of fragments decreases gradually. At the impact pressure of 0.2 MPa, the mudstone samples reveal no obvious macroscopic failure patterns. At the impact pressure of 0.25 MPa, the mudstone samples feature macroscopic cracks and break into two large blocks. At the impact pressure of 0.60 MPa, the mudstone samples break into many small-size blocks. This phenomenon shows that with a further increase in the impact pressure, the distribution of cracks inside the mudstone samples increases, the cracks’ width gradually increases, and the cracks span the entire sample volume. In response to continuous stress, the cracks in the samples further develop and lead to the onset of cross, tensile failure, as well as compression shear failure.

#### 3.3.2. Cyclic-Impact Scenario, Fixed Impact Pressure

**Figure 11:**
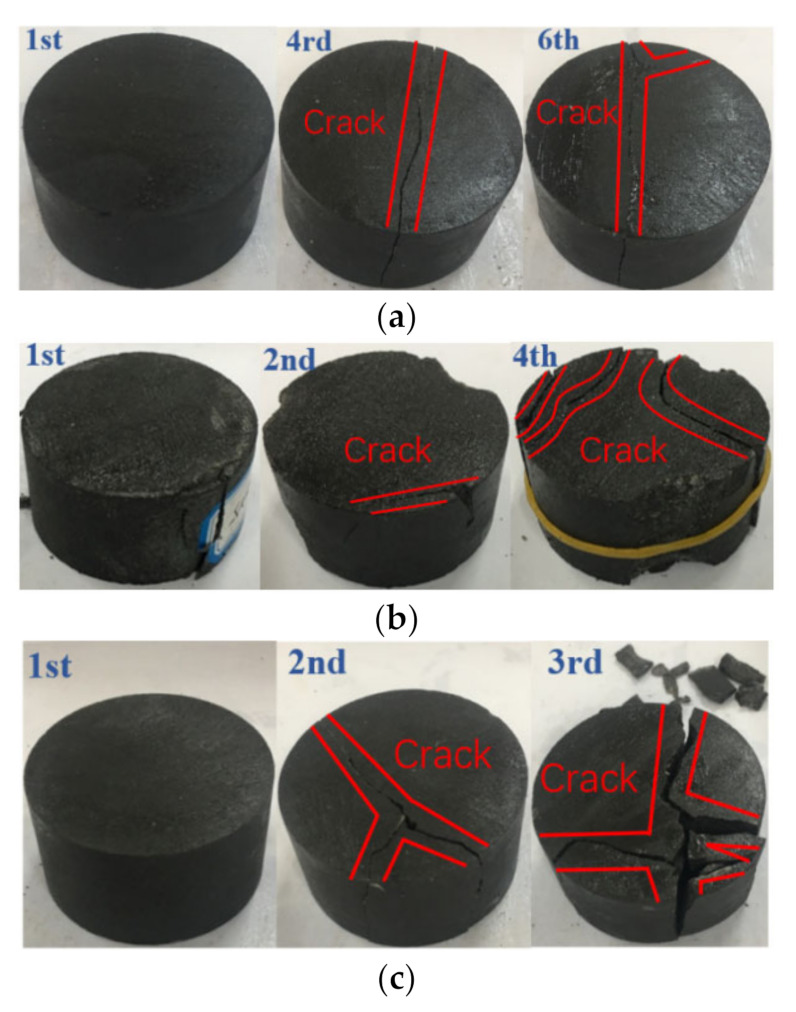
Failure morphology of the mudstone samples subjected to cyclic-impact loads: (**a**) 0.15 MPa; (**b**) 0.175 MPa; (**c**) 0.20 MPa.

The failure morphology of the mudstone samples subjected to cyclic impacts at fixed impact pressures is shown in Figure 11. As the impact pressure increases, the number of impacts until macroscopic failure decreases, and the samples mainly exhibit tensile failure in response to the cyclic-impact loading. For the impact pressure of 0.15 MPa, the mudstone sample remains basically intact after the first impact. As the number of impacts increases, the internal damage to the mudstone sample continues to accumulate, and microscopic cracks are generated and propagate through the sample. Finally, the sample splits into three pieces along the axial loading direction. For the impact pressure of 0.20 MPa, cracks appear on the surface of the mudstone sample after the second impact. During the third impact, the internal damage to the mudstone sample becomes serious, the distribution of microscopic cracks increases, the propagation rate increases, the cracks’ width gradually increases, and they propagate through the sample. The bearing capacity of the sample decreases rapidly, the degree of fragmentation increases, and the annular failure surface covers the sample along the axial loading direction.

## 4. Establishment and Discussion of Dynamic Damage Constitutive Model 

### 4.1. Rock Dynamic Strength Criteria

The above experimental studies demonstrate an obvious “rate effect” in rock samples subjected to impact loads. Therefore, the strain rate is introduced into the static yield function, for developing the rock dynamic yield criterion [29,30,31,32].

The rock static Drucker–Prager criterion is expressed in terms of the first invariant of the stress tensor $I_{1}$ and the second invariant of the stress deviation $J_{2}$, as follows:

(1) $$a_{0}I_{1}+\sqrt{J_{2}}=k$$

(2) $$I_{1}=\sigma_{1}+\sigma_{2}+\sigma_{3}$$

(3) $$J_{2}=\frac{1}{6}\left[{\left(\sigma_{1}-\sigma_{2}\right)}^{2}+{\left(\sigma_{2}-\sigma_{3}\right)}^{2}+{\left(\sigma_{1}-\sigma_{3}\right)}^{2}\right]$$

(4) $$a_{0}=\frac{\sin\varphi }{\sqrt{9+3\sin^{2}\varphi }}$$

(5) $$k=\frac{3c\cos\varphi }{\sqrt{9+3\cos^{2}\varphi }}$$

where c,φ are the static cohesion and internal friction angle of the rock sample, respectively, $a_{0},k$ are the material parameters, respectively, and $\sigma_{1},\sigma_{2},\sigma_{3}$ are the first, second, and third principal stresses of the rock sample, respectively.

The results by Zhao et al. [33,34] and by Song [35] show that a change in the rock dynamic strength in response to a dynamic load is mainly owing to a change in its cohesion, while the internal friction angle changes insignificantly. Thus, the influence of the change in the internal friction angle on the rock dynamic strength can be ignored. The following relationship between the rock dynamic cohesion and static cohesion can be formulated:

(6) $$\frac{c_{d}}{c_{s}}=1.0+\beta \mathrm{lg}\frac{\overset{•}{\varepsilon_{d}}}{\overset{•}{\varepsilon_{s}}}$$

where $c_{d},c_{s}$ are the dynamic cohesion and static cohesion parameters of the rock sample, respectively, ${\overset{•}{\varepsilon }}_{d},{\overset{•}{\varepsilon }}_{s}$ are the dynamic strain rate and static strain rate of the rock sample, respectively, and β is the material constant.

Substituting Equation (6) into the static Drucker–Prager criterion, the rock dynamic strength criterion reflecting the rate effect is obtained as follows:

(7) $$a_{0}I_{1}+\sqrt{J_{2}}=k\left(\overset{•}{\varepsilon }\right)$$

(8) $$k\left(\overset{•}{\varepsilon }\right)=k\cdot \left(1.0+\beta \mathrm{lg}\frac{{\overset{•}{\varepsilon }}_{d}}{\overset{•}{\varepsilon_{s}}}\right)$$

For the one-dimensional stress state, $\sigma_{2}=\sigma_{3}=0$, and the expression for the rock dynamic strength criterion becomes:

(9) $$\left(a_{0}+\frac{1}{\sqrt{3}}\right)\sigma_{1}-k\cdot \left(1.0+\beta \mathrm{lg}\frac{\overset{•}{\varepsilon_{d}}}{\overset{•}{\varepsilon_{s}}}\right)=0$$

The above shows that in the proposed rock dynamic strength criterion the rock dynamic strength nonlinearly depends on the strain rate.

### 4.2. Establishment of the Dynamic Damage Constitutive Model of Mudstone

#### 4.2.1. Dynamic Damage Model

Basic assumptions:

(1) Mudstone is isotropic;
(2) On the microscopic level, mudstone obeys Hooke’s law before damage;
(3) On the microscopic level, the element strength of mudstone is described by a normal distribution.

The rock sample under a dynamic load is abstracted into damaged and undamaged materials, and the load on the rock sample is borne by undamaged materials. According to Lemaitre’s [36] strain equivalence principle, the damage constitutive relationship for a rock sample can be obtained by replacing the nominal stress in the material constitutive relationship with the effective stress, as follows:

(10) $$\sigma_{i}=\sigma_{i}^{*}\left(1-D\right)\begin{array}{cc} & \left(i=1,2,3\right) \end{array}$$

where $\sigma_{i}$ is the nominal stress of rock, D is the damage variable, and $\sigma_{i}^{*}$ is the effective stress of the rock sample.

According to the above basic assumptions, the stress–strain relationship of undamaged materials obeys Hooke’s law; then, for the one-dimensional stress state:

(11) $$\sigma_{i}^{*}=E\varepsilon_{i}$$

where E is the dynamic elastic modulus of rock, $E=\frac{\sigma_{2}-\sigma_{1}}{\varepsilon_{2}-\varepsilon_{1}}$, and subscripts 1 and 2 correspond to the two points of 40% and 60% of the peak value of the dynamic stress–strain curve, respectively.

#### 4.2.2. Statistical Damage Evolution Model

Based on the basic assumption that on the microscopic level the element strength of mudstone follows a normal distribution, the probability density function of the microscopic element strength of mudstone is given as follows:

(12) $$P\left(F\right)=\frac{1}{S_{0}\sqrt{2\pi }}\exp\left[-\frac{1}{2}{\left(\frac{F-F_{0}}{S_{0}}\right)}^{2}\right]$$

where F is the strength parameter of the mudstone microscopic elements, while $F_{0}$ and $S_{0}$ are the normal distribution’s mean and spread parameters.

For a gradually increasing load, the number of failing microscopic elements in the rock sample is:

(13) $$N_{f}=N\int P\left(F\right)dF=N\int \frac{1}{S_{0}\sqrt{2\pi }}\exp\left[-\frac{1}{2}{\left(\frac{F-F_{0}}{S_{0}}\right)}^{2}\right]dF$$

where $N_{f}$ is the number of failing microscopic elements, and N is the total number of microscopic elements.

The statistical damage variable of mudstone is the ratio of the failed microscopic elements to the total number of microscopic elements:

(14) $$D=\int_{-\infty }^{F}P\left(x\right)dx=\int_{-\infty }^{F}\frac{1}{S_{0}\sqrt{2\pi }}\exp\left[-\frac{1}{2}{\left(\frac{x-F_{0}}{S_{0}}\right)}^{2}\right]dx$$

This can be simplified as:

(15) $$D=\varphi \left(\frac{F-F_{0}}{S_{0}}\right)$$

where $\varphi \left(\frac{F-F_{0}}{S_{0}}\right)$ is the standard normal distribution function.

The above expression shows that the damage variable is related to the micro-element strength of the rock sample, and the strength of the rock sample’s micro-elements is related to its stress state. Based on the above rock dynamic strength criterion, a rock dynamic micro-element strength measurement method is proposed, which is expressed as follows:

(16) $$F=f\left(\sigma^{*}\right)=\left(a_{0}+\frac{1}{\sqrt{3}}\right)\sigma_{1}^{*}-k\cdot \left(1.0+\beta \mathrm{lg}\frac{\overset{•}{\varepsilon_{d}}}{\overset{•}{\varepsilon_{s}}}\right)$$

Substituting Equation (15) and Equation (11) into Equation (10), we obtain:

(17) $$\sigma_{d}=E\varepsilon \left(1-D\right)=E\varepsilon \left[1-\varphi \left(\frac{F-F_{0}}{S_{0}}\right)\right]$$

The above equation is the dynamic damage constitutive model of mudstone under an impact load. The rock micro-element strength measurement method adopted in the model reflects the dynamic deformation characteristics of rock and the rate effect of dynamic strength, and can better characterize the nonlinear relationship between dynamic stress and strain in the process of rock dynamic deformation.

### 4.3. Identification of Model Parameters

According to the dynamic damage constitutive equation of mudstone, the key to establishing the constitutive model is the identification of the model parameters $F_{0}$ and $S_{0}$. Because the peak stress $\sigma_{f}$ and peak strain $\varepsilon_{f}$ of the dynamic stress–strain curve are easy to obtain from impact tests, according to the extreme value characteristics of the rock dynamic full stress–strain curve, the two boundary conditions are determined according to the extreme value method of multivariate functional analysis, as follows:

(a) when $\varepsilon =\varepsilon_{f}$, $\sigma =\sigma_{f}$
(b) ${\left.\frac{d\sigma }{d\varepsilon }\right|}_{\begin{array}{c} \sigma =\sigma_{f}\\ \varepsilon =\varepsilon_{f} \end{array}}=0$

Substituting condition (a) into Equation (17), we obtain:

(18) $$\sigma_{f}=E\varepsilon_{f}\left[1-\varphi \left(\frac{F_{f}-F_{0}}{S_{0}}\right)\right]$$

From Equation (18), we obtain:

(19) $$\varphi \left(X\right)=\varphi \left(\frac{F_{f}-F_{0}}{S_{0}}\right)=1-\frac{\sigma_{f}}{E\varepsilon_{f}}$$

From Equation (19), we obtain:

(20) $$X=\frac{F_{f}-F_{0}}{S_{0}},\;\mathrm{then},\;F_{0}=F_{f}-XS_{0}$$

By taking the derivative of Equation (18):

(21) $$\frac{d\sigma }{d\varepsilon }=E\left(1-D\right)-E\varepsilon \cdot P\left(F\right)\left(\frac{\partial F}{\partial \varepsilon }\right)$$

According to condition (b):

(22) $$E\left(1-D\right)-E\varepsilon \cdot P\left(F\right)\left(\frac{\partial F}{\partial \varepsilon }\right)=0$$

where:

(23) $$P\left(F_{f}\right)=\frac{1}{S_{0}\sqrt{2\pi }}\exp\left[-\frac{1}{2}{\left(\frac{F_{f}-F_{0}}{S_{0}}\right)}^{2}\right]$$

By taking the derivative of Equation (16):

(24) $$\frac{\partial F}{\partial \varepsilon }=\left(a_{0}+\frac{1}{\sqrt{3}}\right)E$$

Substituting Equations (23) and (24) into Equation (22), we obtain:

(25) $$P\left(F_{f}\right)=\frac{1-\varphi \left(X\right)}{E\varepsilon_{f}\left(a_{0}+\frac{1}{\sqrt{3}}\right)}$$

From Equation (23), we obtain:

(26) $$S_{0}=\exp\left\{-\frac{1}{2}X^{2}-\ln\left[\sqrt{2\pi }P\left(F_{f}\right)\right]\right\}$$

In conclusion, for the dynamic impact compression test, the values of $S_{0}$ and $F_{0}$ can be determined by substituting Equations (20) and (26) according to the peak point $\left(\sigma_{f},\varepsilon_{f}\right)$ of the dynamic stress–strain curve, elastic modulus E, internal friction angle φ, and strain rate $\overset{•}{\varepsilon }$.

### 4.4. Verification of the Constitutive Model

**Figure 12:**
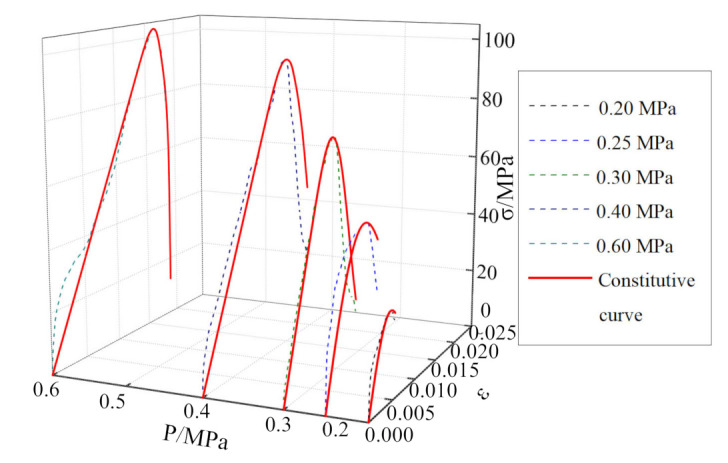
Comparison between the single-impact test results and the theoretical results for mudstone.

**Figure 13:**
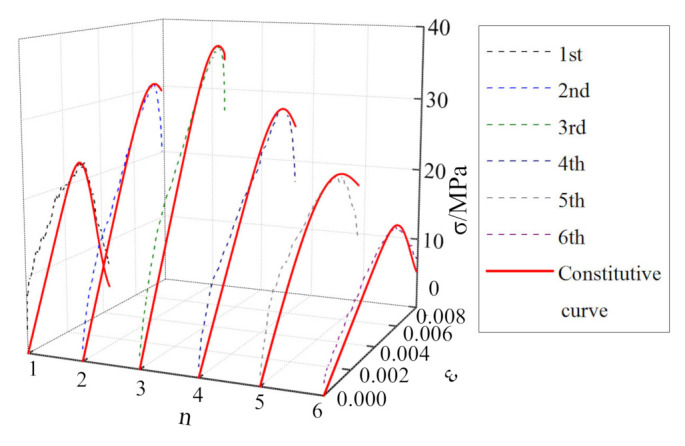
Comparison between the 0.15 MPa-pressure cyclic-impact test results and the theoretical results for mudstone.

**Figure 14:**
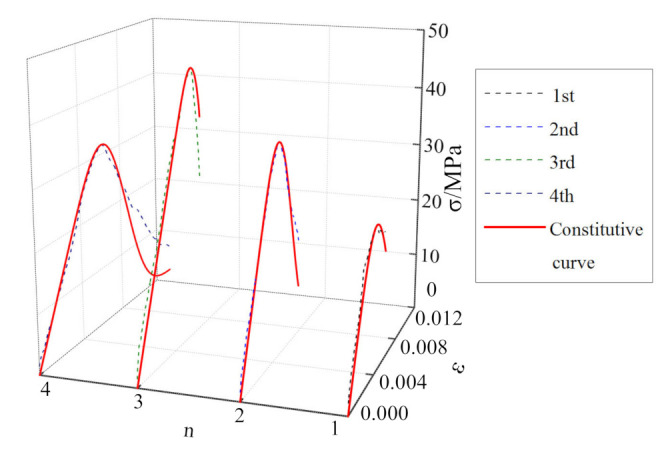
Comparison between the 0.175 MPa-pressure cyclic-impact test results and the theoretical results for mudstone.

**Figure 15:**
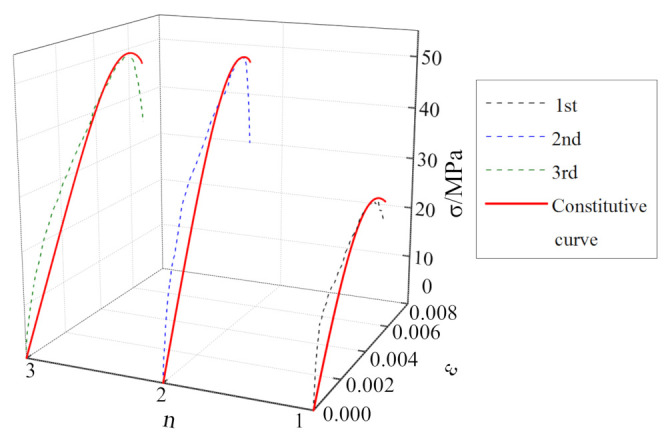
Comparison between the 0.20 MPa-pressure cyclic-impact test results and the theoretical results for mudstone.

To verify the applicability and rationality of the mudstone dynamic damage constitutive model proposed in this study, parameter identification was carried out according to the single impact compression, the cyclic impact compression test data, respectively, as shown in Table 3 and Table 4. The model curve was compared with the test curve, for different impact pressures, and the results are shown in Figure 12, Figure 13, Figure 14 and Figure 15.

It can be seen from Figure 12, Figure 13, Figure 14 and Figure 15 that, although there is some discrepancy between the model curve and the test curve, the overall concordance is high. For example, the model curve cannot accurately reflect fluctuations in the test curve, especially in the initial loading stage. Under the action of a single-impact load (Figure 12), there is a certain discrepancy between the model curve and the test curve for small impact pressures. This phenomenon is mainly owing to the large dispersion of mudstone failures across impact loads, the stochasticity associated with the underlying normal distribution, and the loading pressure’s instability. As the impact pressure increases, the concordance between the test and model curves gradually increases, and the relative standard deviation is only 9% for the impact pressure of 0.6 MPa. It can be seen from Figure 15 that for the cyclic-impact pressure of 0.20 MPa, there is a small discrepancy between the test and model curves after the first impact, while the concordance increases after the second and third impacts, and the relative standard deviation after the third impact is only 15%. In conclusion, the model curve can better reflect the relationship between the dynamic strength, strain, and strain rate for mudstone, which validates the rationality of the dynamic damage constitutive model of mudstone for single- and cyclic-impact loading scenarios.

## 5. Conclusions

(1) According to the stress–strain curves under impact loads, in the single-impact load scenario, the peak stress and peak strain of the studied mudstone samples gradually increased with increasing impact pressure, and the peak stress and the average strain rate were nonlinearly and positively correlated. In the cyclic-impact loading scenario with fixed impact pressure, the peak stress of the mudstone samples increased first and then decreased with the number of impacts, while the peak strain increased gradually with the number of impacts;
(2) Through the analysis of the failure mode of mudstone under impact loads, it can be seen that in the single-impact load scenario, as the impact pressure increased, the fragmentation of the mudstone samples gradually decreased, and the number of broken blocks gradually increased. In the cyclic-impact load scenario, the failure mode of the mudstone samples changed significantly with the increase in impact times. There was no obvious macroscopic failure mode during the previous impact tests, and the tensile failure occurred due to the penetrating cracks of the sample in the last impact;
(3) Based on the rock dynamic strength criterion, combined with the statistical damage theory, a dynamic damage constitutive model of mudstone was established, to describe the mudstone response to various impact load scenarios. The model behavior agreed well with the corresponding experimental results, validating the rationality of the model established in this study. The model provides a theoretical basis for the future stability analysis of roadway-surrounding rock subjected to dynamic loads.

## Figures and Tables

**Table 1:** Results of single-impact tests.

Number	*L*/mm	*D*/mm	*ρ*/kg·m^−3^	Wave Velocity m/s	*P*/MPa	$\overset{•}{\varepsilon }$	$\varepsilon_{f}$	$\sigma_{f}$
1	25.98	49.7	2562	3821	0.20	30.00	0.0042	28.17
2	23.48	49.5	2575	4891	0.25	58.49	0.0073	50.53
3	24.74	49.43	2577	5623	0.30	81.34	0.0088	74.75
4	23.57	49.28	2576	4907	0.40	108.70	0.0149	94.95
5	25.32	49.36	2566	4522	0.60	111.17	0.0165	102.69

Notes: *L* is the length of the sample, *D* is the diameter of the sample, *ρ* is the sample density, *P* is the impact pressure, $\overset{•}{\varepsilon }$ is the average strain rate, $\varepsilon_{f}$ is the dynamic peak strain, and $\sigma_{f}$ is the dynamic peak stress.

**Table 2:** Results of cyclic-impact tests.

Number	*L*/mm	*D*/mm	*ρ*/kg·m^−3^	Wave Velocity m/s	*P*/MPa	n	$\overset{•}{\varepsilon }$	$\varepsilon_{f}$	$\sigma_{f}$
1	25.24	49.7	2537	3712	0.15	1	23.74	0.0028	22.45
						2	30.48	0.0042	32.67
						3	19.59	0.0048	37.81
						4	34.85	0.0054	29.31
						5	39.01	0.0055	20.83
						6	35.56	0.0056	14.39
2	25.16	49.77	2547	3310	0.175	1	48.54	0.0040	24.49
						2	53.54	0.0046	35.68
						3	35.54	0.0059	45.72
						4	63.87	0.0062	31.60
3	25.98	49.7	2562	3821	0.20	1	30.00	0.0042	28.17
						2	35.70	0.0050	51.18
						3	36.11	0.0060	49.09

Note: n is the number of cyclic impacts.

**Table 3:** Parameters of the single-impact dynamic constitutive model of mudstone.

Number	P/MPa	E/GPa	φ/°	c/MPa	$\overset{•}{\varepsilon_{f}}$	$S_{0}$	$F_{0}$	β
1	0.20	11.0	29	21.47	39.95	21.74	28.04	−0.045
2	0.25	12.2	29	21.47	67.42	45.21	59.63	−0.032
3	0.30	10.1	29	21.47	68.87	18.10	45.82	0.177
4	0.40	7.1	29	21.47	93.97	13.17	45.34	0.271
5	0.60	6.5	29	21.47	77.89	7.71	38.81	0.400

**Table 4:** Parameters of the cyclic-impact dynamic constitutive model of mudstone.

Number	P/MPa	n	$\overset{•}{\varepsilon_{f}}$	E/GPa	$S_{0}$	$F_{0}$	β
1	0.15	1	23.98	9.0	3.91	14.99	−0.080
		2	21.79	9.4	9.13	20.33	−0.006
		3	12.47	13.0	9.01	23.70	0.013
		4	32.20	6.5	7.84	18.35	−0.017
		5	42.74	6.1	12.77	19.62	−0.073
		6	42.12	3.5	2.33	8.68	−0.097
2	0.175	1	69.46	7.9	11.30	20.85	−0.054
		2	66.01	8.2	9.24	21.14	0.020
		3	29.16	8.5	6.33	23.60	0.044
		4	73.40	6.2	7.95	18.20	0.003
3	0.20	1	38.80	11.0	21.74	28.04	−0.046
		2	30.75	12.0	26.44	45.55	0.045
		3	30.57	10.5	17.15	36.53	0.054

## Data Availability

The data used to support the findings of this study are available from the corresponding author upon request.
